# Therapeutic Interventions into Innate Immune Diseases by Means of Aptamers

**DOI:** 10.3390/pharmaceutics12100955

**Published:** 2020-10-11

**Authors:** Farzana Yasmeen, Hana Seo, Nasir Javaid, Moon Suk Kim, Sangdun Choi

**Affiliations:** Department of Molecular Science and Technology, Ajou University, Suwon 16499, Korea; farzana892019@ajou.ac.kr (F.Y.); cos159159@ajou.ac.kr (H.S.); nasirjavaid@ajou.ac.kr (N.J.); moonskim@ajou.ac.kr (M.S.K.)

**Keywords:** innate immune system, pattern recognition receptor, aptamer, targeted therapy

## Abstract

The immune system plays a crucial role in the body’s defense system against various pathogens, such as bacteria, viruses, and parasites, as well as recognizes non-self- and self-molecules. The innate immune system is composed of special receptors known as pattern recognition receptors, which play a crucial role in the identification of pathogen-associated molecular patterns from diverse microorganisms. Any disequilibrium in the activation of a particular pattern recognition receptor leads to various inflammatory, autoimmune, or immunodeficiency diseases. Aptamers are short single-stranded deoxyribonucleic acid or ribonucleic acid molecules, also termed “chemical antibodies,” which have tremendous specificity and affinity for their target molecules. Their features, such as stability, low immunogenicity, ease of manufacturing, and facile screening against a target, make them preferable as therapeutics. Immune-system–targeting aptamers have a great potential as a targeted therapeutic strategy against immune diseases. This review summarizes components of the innate immune system, aptamer production, pharmacokinetic characteristics of aptamers, and aptamers related to innate-immune-system diseases.

## 1. Introduction

The immune system consists of several biological processes and structures within an organism that protect it against different pathogens, including fungi, bacteria, parasites, and viruses [1]. This system of protection from foreign microorganisms is classified into two fundamental types: innate and adaptive. These types can be distinguished by the time taken for the response, effector cells, and selectivity of their response to various groups of foreign microorganisms. Innate immunity mounts a nonspecific response to potentially noxious foreign objects, whereas the adaptive immune system has an immense level of specificity and memory. The innate immune system represents the very first line of defense against pathogens. Therefore, a fast response emerges and is incapable of creating immunological memory. It comprises four categories of boundaries, which are named physiological (e.g., low pH and temperature), anatomic (e.g., mucous membranes and the skin), phagocytic (e.g., blood neutrophils, monocytes, and tissue macrophages), and serum inflammatory proteins [2]. 

The innate immune system consists of both constitutive and inducible elements and utilizes a wide range of recognition and effector processes. The innate immune system’s role in the initiation and consequences of an adaptive immune response was well established in the last few years. Constitutive mechanisms are those processes that are active continuously and are not strongly modulated irrespective of the presence or absence of an infection. For example, the epithelial lining provides constant protection to the microbial flora of the skin and genital and intestinal tracts. In comparison, inducible mechanisms of the innate immune system include the production of various cell mediator molecules that help to eliminate microorganisms by upregulating effector molecules. Cell mediators are produced as a result of an encounter with microorganisms and offer less specific immune responses than do antibodies, produced by the adaptive immune system. The leading mechanism behind this type of immune response is the “pattern recognition” of conserved molecular patterns that are essential structural elements of microorganisms. One of the best examples of pathogen-associated molecular patterns (PAMPs) is the glycolipid component of the outer membrane of gram-negative bacteria, called lipopolysaccharide (LPS) [3]. Additionally, the innate immune system can respond to the pattern of some host cells (that undergo cell death) by detecting specialized molecules called damage-associated molecular patterns (DAMPs). They represent various classes of protein and nonprotein molecules. In this way, the response to DAMPs is considered an indirect response to microbial infection; alternatively, it can be started by tissue damage, such as sterile inflammation or ischemia [4,5].

## 2. Representative Components of Innate Immunity

The breach of the host immune system by pathogenic microorganisms generates an array of immune reactions through the synergy between the diversified cluster of pathogen-based virulence factors and defensive immune processes of the host. The host–pathogen encounter usually launches immune reactions via identification of conserved molecular structures known as pathogen-associated molecular patterns (PAMPs) [6]. Active recognition of a PAMP immediately elicits an immune response in the host by stimulating multiplex signaling pathways that climax in the inflammatory responses regulated by numerous chemokines and cytokines, which consequently promote the elimination of the harmful microorganism carrying the PAMP, such as viral double-stranded ribonucleic acid (RNA) and lipopolysaccharides (LPS) [7,8,9]. Moreover, the innate immune system expands efficient defense against pathogenic microbes by initiating adaptive immunity, which involves immunological memory and is long-lasting. Adaptive immunity is characterized by the formation of antigen-specific T and B lymphocytes via gene rearrangement [10].

**Figure 1:**
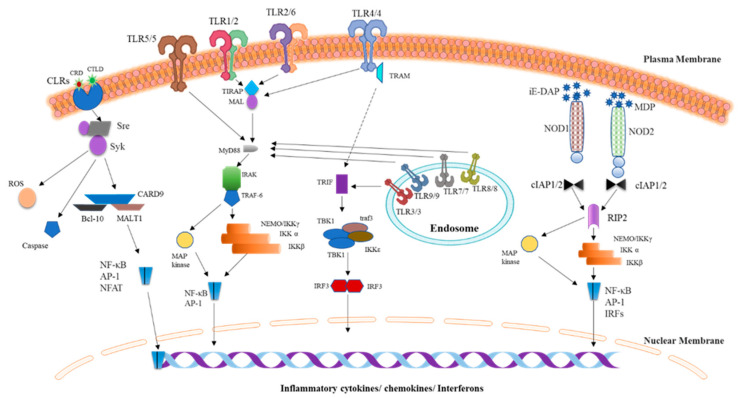
Pattern recognition receptor (PRR) signaling pathways. Three classes of PRR families such as TLRs, nucleotide oligomerization domain (NOD)-like receptors (NLRs), and C-type Lectin Receptors (CLRs) play a critical role in the detection of microbial products or endogenous danger signals with subsequent induction of the innate immune responses that ultimately drive the expression of proinflammatory cytokines. TLRs are classified into two groups depending upon their location: the plasma membrane or endosomes. TLRs are triggered by an encounter with their respective ligands, and then dimerization occurs (homo- or heterodimer formation), and downstream signaling is started via an interaction with cytoplasmic adaptor molecules MyD88, Toll Interleukin-1 Receptor (TIR) domain–containing adaptor inducing interferon β (TRIF), TRAM, and/or TIR domain-containing Adaptor Protein (TIRAP) (MAL). NLR proteins NOD1 and NOD2 are cytosolic PRRs that recognize bacterial γ-d-glutamyl-meso-diaminopimelic acid peptidoglycans (iE-DAP) and muramyl dipeptide (MDP), respectively, then recruit common adaptor molecule RIP2 through CARD–CARD interactions and undergo RIP2 phosphorylation followed by a launch of downstream signaling. CLRs, including dectin-1 and dectin-2, initiate the downstream signaling through Syk- and Raf-1–dependent pathways, which leads to the production of reactive oxygen species, caspase activation, and NF-κB stimulation via the CARD9/Bcl-10/MALT1 complex. The signaling pathways triggered by these three PRRs mainly cause the production of inflammatory cytokines, chemokines, or interferons. Abbreviations: AP-1, activated protein 1; IKK, inhibitor of κB kinase; IL, interleukin; IRAK, interleukin receptor–associated kinase; IRF, interferon response factor; IκB, inhibitor of κB; MAL, MyD88 adaptor–like; MAP, mitogen-activated protein; MyD88, myeloid differentiation primary response 88; NEMO, NF-κB essential modulator; NF-κB, nuclear factor-κB; RIP, receptor-interacting protein; TAB, TAK1-binding protein; TAK1, transforming growth factor β–activated kinase 1; TBK1, TANK-binding kinase 1; TLR, Toll-like receptor; TRAF, tumor necrosis factor receptor (TNFR)-associated factor; TRAM, TRIF-related adaptor molecule; TRIF, TIR domain–containing adaptor inducing interferon β.

The innate immune system identifies pathogenic microbes by a defined number of germline-encoded receptors called pattern recognition receptors (PRRs). They possess some common attributes. First, they recognize specific microbial patterns, PAMPs, which are most evolutionarily and conserved among microbes and, thus, are hard to change. Second, PRRs are nonclonal and evolutionary conserved units which are specific to the germline cells with no immunological memory. Various PRRs for unique PAMPs have specific expression patterns; switch on specialized signaling pathways; and start distinct antipathogen responses. The innate immune system mainly consists of physical (skin and mucous membranes), chemical (reactive oxygen species and antimicrobial peptides), and cellular defenses (neutrophils, monocytes, and macrophages) as well as cellular immune components and mediators including cytokines and the complement system [1,11]. The primary purpose of the innate immune system is to halt the entry of pathogens into the human body by utilizing physical and chemical elements of the body’s defense. It eliminates pathogens by means of innate cytotoxic and phagocytic mechanisms with subsequent switching to an adaptive immune response via antigen presentation to T and B cells [12,13]. Various classes of PRRs along with their signaling pathways have been reported, including Toll-like receptors (TLRs), nucleotide-binding oligomerization domain–like receptors (nucleotide oligomerization domain (NOD)-like receptors; NLRs), and C-type Lectin Receptors (CLRs) (Figure 1). Along with these central innate immune cell receptors, other receptors act as a bridge between the innate and adaptive immune system; these are called “costimulatory receptors.” To effectively perform the innate immune function while clearing the pathogenic microorganism from the body, a plasma protein class called “complement system” plays a huge role. While another small plasma protein class called “cytokines” are secreted by specific cells that have a unique effect on the interactions and communications between cells and fall into the category of both pro-inflammatory and anti-inflammatory cytokines. The TLRs, costimulatory molecules, complement system, and cytokines fall into the category of innate immune components targeted by DNA or RNA aptamers for therapeutic applications and are used in different diseases. The following section will discuss these innate immune components in detail.

### 2.1. TLRs

TLRs are extensively studied and evolutionarily conserved proteins that detect PAMPs. They were initially identified in *Drosophila melanogaster* as an essential gene because of its vital role in ontogenesis and immunological effects against fungal infections [14]. To date, 10 TLR family members have been identified in humans (TLR1 to TLR10) [4]. They are type I integral membrane glycoproteins characterized by their (1) extracellular domains containing varying numbers of leucine-rich repeat (LRR) motifs that are required for PAMP recognition and (2) a cytoplasmic signaling domain homologous to that of interleukin 1 receptor (IL-1R), termed the Toll/IL-1R homology (TIR) domain, which is essential for the activation of downstream signaling. The TIR domain interacts with multiple adaptor molecules and brings about the activation of nuclear factor (NF)-κB through the signal transmission that culminates in the synthesis of proinflammatory cytokines [15]. Among TLRs, TLR1, TLR2, TLR4, TLR5, and TLR6 are mainly located on the surface of the cell and detect PAMPs from fungi, bacteria, and protozoa, whereas TLR3, TLR7, TLR8, and TLR9 are exclusively expressed within endocytic compartments and primarily recognize nucleic acids from various bacteria [16]. Diverse TLRs exclusively detect specific DAMPs and PAMPs [17]. TLR2 forms heterodimers with either TLR1 or TLR6, where TLR1 or TLR2 detects triacyl lipopeptides, while TLR2 or TLR6 specifically interacts with diacyl lipopeptides. TLR3 has high specificity for RNA ligands (double-stranded) that are products of viral replication at various stages. TLR4 recognizes LPS, i.e., the cell wall component of gram-negative bacteria; LPS requires an interaction with coreceptor MD2 to bind to TLR4. TLR5 identifies bacterial-flagellin–based ligands by its extracellular homodimeric domain. Both TLR7 and TLR8 respond to single-stranded RNA, whereas TLR9 interacts with CpG motif–containing ligands [17]. TLRs switch on similar signaling components that are utilized for IL-1R signaling [18]. Signaling through TLRs proceeds essentially through a well-described pathway in which various receptor-binding domains (TIR domains) transmit a signal through adapter molecules such as MyD88, TRIF (TICAM-1), TIRAP (MAL), and TRAM [10]. These adaptor molecules stimulate specific transcription factors like IRF3/7, nuclear factor κB (NF-κB), and mitogen-activated protein kinases (MAPKs) to induce the expression of type I interferons and proinflammatory cytokines. All TLRs, except TLR3, engage MyD88, and launch MyD88-dependent signaling pathway to cause NF-κB and MAPKs to upregulate proinflammatory cytokines in dendritic cells and macrophages. On the other hand, TLR1, TLR2, TLR4, and TLR6 employ TIRAP to activate MyD88-dependent signaling. TLR3 and TLR4 initiate TRIF-dependent signaling to make NF-κB and IRF3 upregulate type I interferons and proinflammatory cytokines. TLR4 employs TRIF through a complementary adapter molecule, TRAM. Meanwhile, TLR4 triggers the TRIF-dependent signaling pathway together with MyD88 signaling by recruiting all four adapter molecules. First, TLR4 uses TIRAP, which enables MyD88 recruitment to induce MAPK and NF-κB activation. TLR4 is pushed to an endosome through dynamin-dependent endocytosis during TRIF-dependent signal transduction and forms a complex with TRIF and TRAM. This complex initiates TRIF-dependent signaling, which is essential for forcing IRF3 to upregulate a type 1 interferon and the second phase of NF-κB and MAPK stimulation to trigger the production of inflammatory cytokines [19]. In dendritic cells, a protein limited to the endoplasmic reticulum, UNC93B1, plays an integral part in the transport of endosome-localized TLRs, including TLR3, TLR7, and TLR9. Mice that carry a mutation in this protein show absolute absence of all cytokine production after encountering respective PAMPs [20,21,22].

### 2.2. Costimulatory Molecules/Receptors

Costimulatory molecules are categorized into three major groups, namely (i) immunoglobulin (Ig) superfamily, (ii) tumor necrosis factor (TNF) receptor superfamily (TNFR), and the emerging T cell Ig and mucin (TIM) domain family. They cannot activate T cells independently; however, they are crucial to functional naïve T cell response, which ultimately depends upon the consequence of the union of these stimulatory or inhibitory signals [23]. T cells’ activation needs a first signal from the integration of antigenic peptide major histocompatibility complex (MHC) with T-cell antigen receptor (TCR) and a second signal from antigen-independent co-signal, the ’costimulatory signal. Jenkins and Schwartz et al. reported that in the absence of a costimulatory signal, T cells’ TCR-mediated activation comes out in the antigen-specific unresponsiveness a phenomenon called T-cell anergy. Therefore, costimulation is considered to have a central role in regulating the outcome of T-cell contact with the antigen, whether it results in anergy or activation. The essential role of costimulation to regulate the immune response has driven the researchers to study further about it from therapeutic point of view. Earlier studies demonstrated the role of cluster of differentiation (CD)28 receptor for the naive T cells and evaluated the role of B7 family members such as B7-1 (CD80) and B7-2 (CD86) by their respective ligands. The encounter of CD28 and B7-1/B7-2 satisfied many requirements to strengthen the postulates by Lafferty, Schwarz, and colleagues for costimulatory signaling. The interaction between CD28 and B7-1/B7-2 fulfilled many of the requirements for the costimulatory signal postulated by Lafferty, Schwarz, and colleagues. It was illustrated that the CD28 homolog cytotoxic T lymphocyte-associated antigen-4 (CTLA-4) exhibited a greater binding affinity for B7-1 and B7-2 in comparison to CD28 [24]. There was also a consideration that CTLA-4 would also be a stimulatory receptor, but the lethal inflammatory phenotype of CTLA-4 lacking mice (−/−) depicted the inhibitory function of CTLA-4. Moreover, CTLA-4 (−/−) mice exhibited the T-cell costimulatory receptors to convey stimulatory and second inhibitory signal for T-cell responses and indicated that the second inhibitory signals (co-inhibitory) could regulate T-cell tolerance [25].

The first costimulatory molecule to be identified as a member of the TNFR/TNF superfamily was CD154 (also called CD40 ligand) has a critical role in the function of B cells and dendritic cells (DCs). CD40 transduces the signals to dendritic cells (DCs) and B cells along with other cell types, such as tumor cells. Noelle and colleagues discussed the downstream signaling pathways activated by CD40 through tumor necrosis factor receptor associated factor (TRAF) proteins. They also showed the fundamental role of CD4/CD40L interactions in regulating cellular, humoral, and tumor immunity [26]. The members of TNFR/TNF family members, CD27/CD70, CD30/CD30L, OX40/OX40L, 4-1BB/4-1BBL, glucocorticoid-induced TNF receptor (GITR)/GITR ligand (GITRL) provide necessary costimulatory signals. Among them, CD27 is expressed on naive T cells while others are expressed only upon T-cell activation. Recent work suggested that OX40/OX40L and 4-1BB/4-1BBL interactions have a significant role in controlling the balance between effector and T_reg_ responses. Croft et al. discussed that OX40 boost effector T-cell expansion, survival, maintenance, generation, and reactivation of memory T cells [27]. OX40 blocks T_reg_ activity along with antagonizing the generation of inducible T_regs_. Consequently, OX40 stimulates effector and memory T cell responses directly by activating these cells and indirectly by inhibiting T_regs_. Due to the dual-functional capacity of OX40, while blockade can attenuate the inflammation and autoimmunity and stimulation can enhance anti-tumor immunity, it has become an attractive therapeutic target. The primary role of 4-1BB is the survival of activated and memory T cells with particular impact on CD8+ T cells. The 4-1BB signals can cooperate with TCR-induced signals to increase the development and proliferation of response when other costimulatory signals are limited. 4-1BB is expressed on T_regs_, and their ligation could enhance CD8+ T-cell and interferon-γ (IFN-γ)-dependent suppression of CD4+ T-cell responses.

### 2.3. Complement System 

The complement system is a significant part of the innate immune system’s effector mechanism that works as a bridge between innate and acquired immunity. It mainly consists of a collection of proteins that are synthesized in the liver and circulate in the blood plasma and on cell surfaces in the form of inactive precursor zymogens [28]. The complement system plays a vital role in infections and involves a wide range of physiological and pathological processes [29]. There are three known pathways for complement activation: classical, alternative, and lectin pathway.

#### 2.3.1. Classical Pathway

The classical pathway is activated by binding immunoglobulin G (IgG) or IgM antigen/antibody complexes to C1q (first protein of cascade), leading to the activation C1r and, in turn, cleaves C1s. This results in the activation of serine proteases that cause cleavage of C4 and C2. This leads to C4b2a (C3 convertase) development, cleaving the C3 into C3a, and C3b [30]. The C3a will cause the recruitment of the inflammatory cells (anaphylatoxin), and in contrast, C3b binds to the C4b2a complex to formulate C5 convertase (C4b2a3b). Besides, the C5 convertase will activate the formation of the Membrane Attack Complex (MAC), which will form pores in the bacterial membrane leading to its lysis (30). The classical pathway can also be initiated by other danger signals such as viral proteins, C-reactive protein, polyanions, amyloid, and apoptotic cells [31,32,33]. This shows that the classical pathway can be activated without antibodies.

#### 2.3.2. Lectin Pathway

Like the classical pathway, the lectin pathway also leads to the formation of C4bC2aC3 convertase complex in its activation. However, the lectin pathway utilizes members of the collectin family of plasma proteins called mannose-binding lectins (MBLs) and ficolins to identify the ligands’ carbohydrate patterns expressed on the surface of various microorganisms. In addition, the ficolins are homologous to MBL. When MBL or ficolins binds to sugar molecules expressed on pathogens’ surface, MBL-associated serine proteases are activated, i.e., MBL-Associated Serine Proteases (MASP)-1, MASP-2, MASP-3. This, in turn, cleaves the C4 and C2 to generate C4bC2a in a similar way as in the classical pathway [34].

#### 2.3.3. Alternative Pathway

In contrast to the classical and lectin pathways, the alternative pathway is activated at low levels of pathogenic microorganisms in the normal host. This is mainly referred to as a *tickover* mechanism that allows the system to remain primed for a fast and robust activation. The hydrolysis of a thioester bond within C3 initiates the alternative pathway, resulting in a conformational change of the C3 structure. It is referred to as C3(H_2_O), which has similar binding functionality to factor B as C3b. This bound CFB will act as a substrate for serine proteases factor D (CFD). Moreover, the cleavage of CFB by the CFD leads to the formation of alternative pathway C3 convertase C3(H_2_O)Bb process, which is similar to the classical pathway C3 convertase (C4bC2a) and can cleave C3 into C3a and C3b. Moreover, the C3b generation by forming the amplification loop of C3 convertase C3bBb allows complete activation of the alternative pathway in either fluid phase represented by fluid phase C3b or surface bound C3b on the activating surface [35].

### 2.4. Interleukins 

Interleukins are type of cytokines, initially discovered in 1955, secreted by leukocytes and various other types of cells [36]. Interleukin-1 (IL-1) family contains 11 cytokines and 10 receptors that play an essential role in the regulation of immune and inflammatory responses to infections via activation and differentiation of immune cells in addition to maturation, proliferation, migration, and adhesion. Interleukins also show pro-inflammatory and anti-inflammatory activity after binding to high-affinity receptors on the cell surfaces. IL-1 family system is strictly regulated at different points by decoy receptors, antagonists, scavengers, and dominant-negative molecules. The excessive or unchecked activation of the IL-1 family is the leading cause of detrimental and dangerous local or systemic inflammatory, allergic or autoimmune reactions [37]. Therefore, IL-1 family therapies have an enormous beneficial influence in treatment of inflammatory diseases. IL-1 family system is further divided into three sub-families. These include the IL-1 sub-family (IL-1α, IL-1β, and IL-33, IL-1Ra), IL-18 sub-family (IL-18 and IL37), and IL-36 sub-family (IL-36-α, -β, -γ, and IL-38). Among the cytokines, the sub-families are characterized as “pro-inflammatory” and “anti-inflammatory” such as IL-1α, IL-1β, IL-18, IL-33, and IL-1Ra, IL-36Ra, IL-37, IL-38, respectively [38,39,40]. 

Among the IL-1 family, there are seven agonistic ligand molecules (IL-1α, IL-1β, IL-18, IL-33, IL-36-α, -β, and -γ), three receptor antagonists (IL-1Ra, IL-36Ra, and IL-38), and an anti-inflammatory cytokine (IL-37). In the common IL-1 family activation pathway, the receptor chains commonly consist of an extracellular portion having three Ig-like domains except for IL-18 binding protein and TIR8, which have a single Ig domain. The intracellular portion of these receptors is characterized by a TIR domain that conducts the signal through the MyD88 adaptor molecule. The IL-1 family cytokines mediate the intracellular signaling pathway by binding to a primary receptor subunit such as IL-1 RI/IL-1 R1, IL-18 R α /IL-1 R5, IL-1 Rrp2/IL-1 R6, or ST2/IL-1 R4 which subsequently recruits an accessory receptor to form an active receptor complex (such as IL-1 RAcP or IL-18 R β) to activate downstream signaling. IL-1α, IL-1β, IL-18, IL-33, IL-36-α, -β, and -γ activates the intracellular signaling pathway that recruits the NF-κB and AP-1-dependent expression of chemokines, pro-inflammatory cytokines, and secondary mediators of the inflammatory response. Furthermore, other members of the IL-1 family prevent inflammation by acting as antagonists of IL-1 or IL-36 signaling. The IL-1Ra negatively controls the IL-1 signaling pathway by binding to IL-1 RI and inhibits its ability to interact with IL-1α and IL-1β. Likewise, IL-36Ra binds to IL-1 Rrp2 and inhibits IL-36 signaling [41]. Moreover, the cytokine receptor IL-6R and cytokines such as IL-8, IL-10, and IL-11 have been targeted by aptamers for therapeutic applications. Thus, the detailed pathway of these interleukins is described below.

#### 2.4.1. IL-8 Signaling Pathway

IL-8 is a small soluble protein and belongs to the CXC chemokine family [13]. IL-8 was originally identified as a potent chemotactic and neutrophil activator factor secreted by activated macrophages and monocytes [15,16,17]. Moreover, neutrophils, lymphocytes, endothelial cells [21] and fibroblasts, also secrete IL-8 [42,43,44,45]. IL-8 holds the pro-angiogenic property, confirmed by a Glu-Leu-Arg motif that precedes the first N-terminal cysteine residue [46]. The biological activities of IL-8 are mediated by rhodopsin-like guanine-protein-coupled receptors (GPCRs): CXCR1 (IL-8RA) and CXCR2 (IL-8RB). CXCR1 and CXCR2 are characterized by 7-transmembrane-spanning regions, an extracellular N-terminus, and an intracellular C-terminus [47]. CXCR1 is stimulated by IL-8 and granulocyte chemotactic protein-2 (GCP-2)/CXCL6 [48]. CXCR2 can be activated not only by IL-8 but by many other CXC chemokines, for example, neutrophil-activating protein-2 (NAP-2)/CXCL7 and growth-regulated oncogene. Various studies stated that the signaling of IL-8 requires interaction between the N-terminal region of IL-8 and the N-terminal extracellular domain of the receptors [49]. This ligand binding activates the exchange of guanosine diphosphate for guanosine triphosphate on the Gα subunit, which activates the release of this subunit from the receptor and the Gβγ subunits [50]. Subsequently, Gα and Gβγ subunits activate a variety of signaling pathways in different cell types. The major three pathways include phosphatidylinositol 3 kinase/Akt (PI3K/Akt), phospholipase C/protein kinase C (PLC/PKC), and Ras/Raf/extracellular signal-regulated protein kinases 1 and 2 (Erk1/2). Other signaling pathways include focal adhesion kinase, Rho, Rac, and Janus kinase/signal transducer and activator of transcription (JAK/STAT) [51].

#### 2.4.2. IL-10 Signaling Pathway 

The binding of IL-10 to the receptor complex activates the Janus tyrosine kinases, JAK1 and Tyk2, associated with IL-10R1 and IL-10R22. The domain of STAT3 recruitment is shared by other STAT3-recruiting receptors, IL-20R1, IL-22R1, and including gp130 [52]. Homodimerization of STAT3 drives its release from the receptor and translocate the STAT homodimer into the nucleus, where it binds to the STAT-binding promotor region of various genes, e.g., IL-10 itself. In addition, STAT3 stimulates cytokine signaling 3 (SOCS3) suppressor and, consequently, regulates the quality and quantity of STAT activation. SOCS proteins are composed of two primary domains: one is the substrate-binding domain called Src homology 2 (SH2) domain and second Socs box that form a complex with belongings B and C, a cullin and Rbx2, to form an E3 ubiquitin ligase [53]. The roles of STAT3 and SOCS in IL-10 signal transduction have been well established [54]. Moreover, IL-10 activates another pathway, the phosphoinositide 3-kinase (PI3K) pathway. Inhibition of NF-κB by IL-10 well explains many genes that remain unresponsive to IL-10 treatment. Dendritic cell maturation is also inhibited by defective NF-κB activation, decreasing the antigen-presenting cell (APC) function. Moreover, the inhibition of Rel family members, like p65, is an example of IL-10 inhibitory functionality for inflammatory genes, but it activates other genes. It is reported that IL-10 inhibition may not occur in human monocytes at the extent of NF-κB inhibition [55]. The possible causes may include activation of an inhibitory PI3K/AKT and/or inhibition of MAPKs [56]. Therapeutic aptamer for IL-10R has been developed, will be discussed in Section 6.5.

#### 2.4.3. IL-6 Signaling Pathway

Interleukin-6 (IL-6) is a pleiotropic cytokine that plays a significant role in the immune system and various biological functions such as hematopoiesis, inflammation, and oncogenesis by controlling cell survival growth, proliferation, and differentiation [57]. IL-6 receptor is composed of two distinct subunits IL-6Rα (gp80 or CD126), an 80-kDa type I transmembrane protein, and IL-6Rβ (gp130 or CD130), a 130-kDa second signal transmembrane protein. The soluble IL-6R, which is cleaved from the cell membrane, can still bind its ligand IL-6. Interleukin-6 exerts its activity mainly through binding to the cell membrane IL-6 receptor. Upon binding of IL-6 to IL-6R, homodimerization of gp130 is induced and a high-affinity functional receptor complex of IL-6, IL-6R, and gp130 is formed which initiates cellular events including activation of Janus kinase (JAK) kinases and activation of Ras-mediated signaling [58]. Activated JAK kinases phosphorylate and activate STAT transcription factors, particularly STAT3 (Signal Transducers and Activators of Transcription-3) and SHP2 (SH2 (Src Homology-2) domain-containing Tyrosine Phosphatase). Phosphorylated STAT3 then forms a dimer and translocate into the nucleus to activate transcription of genes containing STAT3 response elements. STAT3 is essential for gp130-mediated cell survival and G1 to S cell-cycle-transition signals. Both c-Myc and Pim have been identified as target genes of STAT3 and together can compensate for STAT3 in cell survival and cell-cycle transition. SHP2 links the cytokine receptor to the Ras/Mitogen-Activated Protein (MAP) kinase pathway and is essential for mitogenic activity [59].

Therapeutic aptamers against Interferon γ and TNFα have also been developed. Interferon γ is primarily secreted by activated T cells and natural killer (NK) cells which can promote macrophage activation, mediate antiviral and antibacterial immunity, enhance antigen presentation, and coordinate lymphocyte–endothelium interaction [60]. TNFα plays an important role in various physiological and pathological processes, including cell proliferation, differentiation, apoptosis, and modulation of immune responses and induction of inflammation [61]. The detailed signaling pathways can be checked from their reference papers, respectively.

## 3. Aptamers

Aptamers include single-stranded DNA or RNA molecules adopting a robust three-dimensional (3D) conformation that can potentially bind to diverse groups of molecular targets with excellent specificity and affinity [62]. The assumption of 3D structure both in vitro and in vivo with increased specificity and selectivity to their target molecules is the reason for aptamers to be called as “chemical antibodies” [63,64,65]. The first aptamer (RNA) was developed in 1990. Two high-affinity RNA aptamers for the T4 DNA polymerase were enriched from an 8-nucleotide random library in an in vitro experiment [66,67]. Ellington named these molecules “aptamers” by combining the Latin word “Aptus,” meaning “to fit,” and the Greek word “meros” meaning a “part.” Aptamers hold the formation of complementary canonical base pairs that is an overwhelming feature and resulting in the formation of double-stranded hairpin DNA or RNA structure [65]. Aptamers have probability to form diverse secondary structures such as a bulge, loop, pseudo-knot, stem, kissing hairpin, or G-quadruplex. Sequentially, the aggregation of these secondary structures gives rise to distinctive 3D structures that are highly specific to their respective targets. The 3D interactions among aptamers, which are considered essential for their binding specificity and affinity, involve hydrogen bonding, electrostatic and hydrophobic interactions, and base stacking, which result in complementary van der Waals forces [68]. These unique 3D interactions generate aptamer–target complexes equivalent to antigen–antibody complexes. The aptamer–target complexes involve equivalent or even better binding specificity and affinity as compared to antigen–antibody complexes [68]. Other than that, aptamers have a strong tendency to distinguish between very similar molecules such as targets carrying distinct functional groups [69], conformational isomers, or mutation of even one amino acid [70]. Over the past three decades, various aptamers have been developed for hundreds of target molecule(s). Aptamers have been developed against numerous such molecular targets as nucleotides, peptides, amino acids, cofactors, aminoglycosides, antibiotics, base analogs, drugs, and diverse proteins of therapeutic significance such as enzymes, immunoglobulins, growth factors, surface receptors, and gene-regulatory factors [66,71]. Antibodies are considered the preferred treatment method in many diseases, but the extensive use of antibodies may cause immune stimulation and toxicity. In this regard, the aptamers utilize a chemical production system and small size that could not be recognized by the immune system. Therefore, there are fewer chances of immunogenicity when aptamers are used as therapeutics [72]. The specialized secondary and tertiary structures of aptamers allow them to recognize various targets with their relevant affinity [73]. The natural presence of four base pairs in aptamers limit their structural conformations; therefore, various modified bases have been introduced to widen the range of structures and nucleotide-protein interactions [74,75]. The overall conformation of nucleic acid is executed by various contributing factors such as phosphodiester backbone flexibility, possible base pairings, noncanonical base pairing resulting from hydrophobic interactions and free energy. Secondary structures occur as an outcome of intramolecular nucleotide pairing while noncanonical base pairs are set up as a result of hydrogen bonds which are further stabilized by polar hydrogen bonds as well as interactions between C-H and O-N groups [76]. The 3D conformation of aptamers is based upon its secondary structure which is directly responsible for their binding affinity and specificity to cognate targets [65]. Moreover, hydrogen bonding, electropositive charge-charge interactions, and van der Waals forces are also involved in the binding interaction of aptamers with their protein targets [68]. Nonetheless, the development of clinically useful therapeutic aptamers has straggled behind the design of therapeutic antibodies. Experiments have also been conducted with aptamers against whole viral particles, whole cancer cells, and pathogenic bacteria [77]. 

## 4. Aptamer Production

The central concept in the use of nucleic acid molecules (aptamers) as therapeutic agents is based upon RNA and DNA molecules’ inherent ability to adopt a folded and unique 3D conformation. It helps to provide a scaffold for binding to the functional groups of their target ligand molecules, which may be proteins or low-molecular-weight compounds such as amino acids. Aptamers bound to the fluorescent dyes are also used as biosensors in different diseases such as cancer to detect cancer cells [78]. To stop the allograft rejection and hypersensitivity reactions, aptamers are produced against autoantibodies [79,80]. In the case of nucleic acids, systematic evolution of ligands by exponential enrichment (SELEX) is considered useful because of specific amplification of high-affinity–selected molecules. There is no difference between RNA and DNA aptamers in terms of their function, but they have their own advantageous properties. DNA aptamers offer highly stable structure with low manufacturing cost, but their stability could be hampered by the nucleases present in biological systems [81]. By contrast, RNA aptamers have stronger intrastrand RNA–RNA interactions due to the presence of additional hydroxyl group at 2′ position of ribose in RNA and predominant single-stranded form [82]. These attributes boost their binding specificity and affinity because of more diverse 3D conformations as compared to DNA aptamers [83]. RNA aptamers have numerous advantages because of their intracellular targeting ability. They could attach directly to extracellular targets to modify their functions, e.g., agonist or antagonist. This feature of RNA aptamers can be utilized to deliver therapeutic agents into specific cell types for the treatment of different human diseases. Furthermore, aptamers can be generated by a high-yield procedure in a bacterial or viral-contamination–free environment [71]. The most effective procedure for the preparation of aptamers is the SELEX technology.

### 4.1. SELEX

This is a gold standard procedure for the preparation of diverse RNA or DNA aptamers [84,85]. Nucleic acid aptamers chosen from a random sequence library by SELEX are capable of binding to a given ligand with unique affinity and a remarkable specificity. The SELEX technique enables the selection or evolution of molecules in a population of random-sequence nucleic acids library by the method of their exponential enrichment [71]. Generally, the procedure starts with compilation of an RNA or DNA library consisting of 20–60 nt random sequences flanked at 5′ and 3′ ends by fixed primers. The fixed part contains a promoter region for an RNA polymerase (T7) and primers for reverse transcription (RT) and polymerase chain reaction (PCR). Theoretically, the initial random library contains up to 10^15^ unique sequences which can offer ample structural variety for recognition of a target with affinity to an extent; this affinity can be further enhanced by applying different modifications. Generally, primers up to 20 nucleotides long are chosen and can be produced with a good yield [86]. 

There are a few differences between DNA and RNA aptamer selection procedures. DNA SELEX is followed by incubation of the DNA library with a target molecule(s), and bound sequences are eluted and recovered after successive reamplification. By contrast, in RNA SELEX, the RNA molecules first undergo RT into double-stranded DNA molecules for successive RNA transcription. For the next selection cycle, the DNA aptamer library goes through the strand separation to obtain new single-stranded DNA molecules that became double-stranded after the amplification. In contrast, RNA SELEX involves in vitro transcription [83]. During the SELEX procedure, nucleic acid molecules can be amplified either by reverse transcriptase-polymerase chain reaction (RT-PCR) or conventional PCR [71]. In case of RNA screening, the PCR amplification method could be utilized as screening method due to the fact that DNA is synthesized from an RNA template with reverse transcriptase, and the RNA is synthesized from a DNA template with RNA polymerase. Thus in vitro selection method would be used for screening of modified nucleic acids [87].

A single nucleotide unit consists of sugar, nitrogenous base, and a phosphate group which could be subjected to chemical modification. In order to make the aptamers as a suitable candidate for therapeutic use various types of chemical analogs are introduced in the aptamer library to insert the modifications, such as: purine analogs are substituted at 7th or 8th positions of the base unit; pyrimidine analogs are substituted at the 5th position and 2′ position of the sugar moiety and phosphate analogs are also inserted in the place of "oxygen" atom. The resulted analogs can offer a relatively suitable substrate for a polymerase reaction. The generation of modified nucleic acid can be affected by chemical structure; position replacement of the modified moiety, and the type of polymerase used. The template DNA would be transcribed by using modified ribonucleoside triphosphates rather than natural triphosphates to synthesize modified RNA. For this purpose, T7 and SP6 RNA polymerases have been used. After completion of the screening process, the selected modified RNA would be reverse transcribed to DNA and then PCR amplification would be done. *Tth* DNA polymerase, with reverse transcription activity, or other types of modified RNA polymerases are ideally used in the reverse transcription. These modified RNAs will function as a template for the reaction catalyzed by those polymerases to yield the corresponding DNA products. Modified deoxynucleoside triphosphates would be used as substrates for the amplification of modified DNA with PCR. In PCR, modified substrates are needed to efficiently incorporate into the extending strand and the produced modified nucleotide strands are required to act as templates for the next thermal cycle. At this point, the incorporation efficiency relies on the type of thermostable DNA polymerase being used in PCR. Commercially available thermostable DNA polymerases for PCR include *Vent(exo)*, KOD Dash and Phusion DNA polymerases derived from *Thermococcus kodakarensis* are well known [88,89]. Moreover, Taq or Tth DNA polymerases are not suitable for modified DNA synthesis and are sensitive to chemical modifications [90,91].

**Figure 2:**
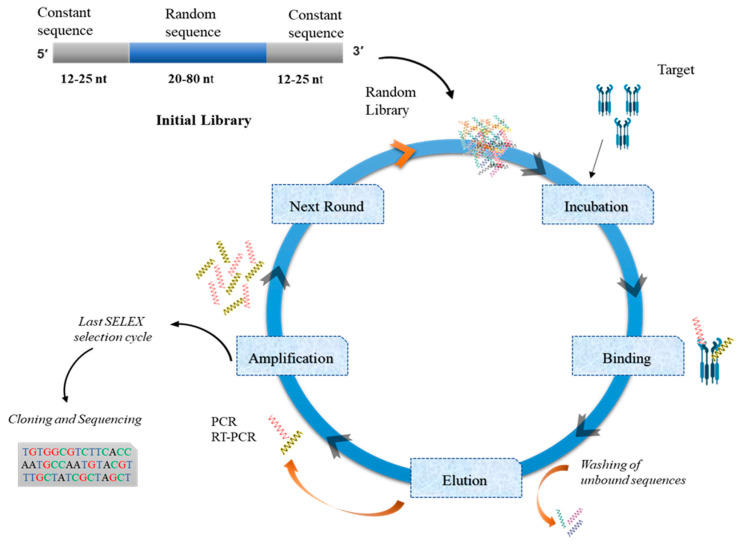
The systematic evolution of ligands by exponential enrichment (SELEX) process. Aptamer selection represents a sequential path as shown in the figure. This evolutionary process starts with the initial random library of oligonucleotides (that contain specified constant and random regions) directed against a target molecule(s). After aptamer–target incubation, unbound sequences are washed off, and bound sequences are eluted. These eluted sequences are amplified repeatedly, and this selection scheme must be repeated for 15 to 25 cycles. Then, final eluted aptamers are subjected to cloning and sequencing.

After repeated aptamer selection rounds, including attachment, portioning, recovery, and reamplification, unique aptamer sequences prevail in the whole library. The final product is obtained via a combination of experimental conditions, e.g., the target-to-library ratio, ionic strength, buffer constituents, binding temperature, elution buffer, pH, and a target’s attributes such as hydrophobicity, charge, and pI. All these factors collectively constitute a unique selection strategy that influences the specificity, affinity, and function of the enriched aptamers [65]. The key considerations to take note of during the design of aptamer libraries are summarized below. For schematic SELEX protocol, see Figure 2.

#### 4.1.1. Randomization

Three types of randomization are applied to the aptamer random-sequence region: partial, segmental, and complete randomization. A partially randomized library of aptamers is selected to find new molecules by considering an existing molecule whose structure, function, and binding affinity for the target molecule are known. In segmental randomization, an agreement is reached between complete and partial randomization in long nucleotide sequences. In complete randomization, aptamer oligonucleotide sequences are selected with either no prior information on the structure or with minimal knowledge about the target or aptamer. The length of the random sequence and of variant sequences flanking the motifs provides the basis for a partially randomized pool and is proportionate to a mutation in each nucleotide at a persistent rate, following a sequence of point mutations close to the natural limit at a much higher speed [92]. 

#### 4.1.2. Random-Region Length 

To develop the aptamer, it is important to specify the length of the random-sequence region. Larger random regions can form a broad range of 3D structures; therefore, it is assumed that a sizable random-sequence region may enable the selection of more efficient aptamers [93]. If the random region is N nucleotides long, it corresponds to 4^N^ variants. A short aptamer having a random-sequence region of 20–50 nucleotides will give rise to a large number of structural and functional variants. Because the initial mass of oligonucleotides for initiating SELEX is always finite, it will practically, as well as naturally, control the number of variants regardless of the theoretical maximum number it corresponds to. Thus, it is practically infeasible and statistically impossible to synthesize an oligonucleotide library that theoretically has the highest variety. On practical grounds, in vitro selection of oligonucleotides is restricted to 4^30^ variant sequences (approximately 10^20^) [92].

#### 4.1.3. Aptamer Library Chemistry 

In terms of the development of aptamers having considerable affinity for the target molecules, the chemistry of the oligonucleotide pool also affects the selection procedure. With the increasing size of a DNA molecule, the maximum number of variants per unit mass (mg) needs to be calculated. In in vitro selection, modified nucleotides (NTPs) are also used to attain the desired pharmacokinetic properties of aptamers. Recombinant RNA polymerase (T7) can incorporate various modified ribonucleotides, e.g., phosphorothioate or 2′-fluoro- and 2′-amino-nucleotides, 2′-chloro and 2′-azido, 2′-fluoro-arabino, 2′-*O*-4′*C*-methylene-*β*-d-ribofuranose moiety, hexitol nucleic acid, α-L-threose, and 4′-thio monomers have been successfully introduced in this manner [86,94,95]. 

One of the most pivotal and challenging steps in the SELEX technique is the separation of bound sequences from unbound sequences because any fault in this step can yield false selection and further amplification of useless ligands. This is the point in the whole SELEX scheme where numerous patents on aptamer production have been coming to naught. Various methods are being adopted depending on the nature of target molecules, target–aptamer interaction, future use of the aptamer, and the likelihood of success. Examples include solid-phase matrix-based segmentation strategies such as chip-based segmentation [96]. The nitrocellulose membrane is a popular matrix used in SELEX owing to its high protein-binding affinity over nucleic acids. In contrast, the nylon membrane favors the binding of negatively charged oligo-nucleotides for blotting applications with nucleic acids, such as a double filter assay. When the mixture of the random library and target protein is passed through a nitrocellulose membrane filter, size exclusivity ensures that larger aptamer–protein complexes are retained on the membrane while small, unbound nucleic acid molecules with weak affinity to the membrane freely flow through [65]. With magnetic beads, the target protein is first immobilized on the beads’ surface, which is then incubated with the random library; the resulting mixture is subjected to magnetic precipitation to recover bound sequences. After desalting and purifying with the ethanol precipitation method, these bound sequences are then amplified via PCR to prepare an enriched oligonucleotide library for the subsequent round of SELEX [97]. In conventional SELEX, the final enriched library is subjected to cloning and Sanger sequencing analysis to detect individual aptamer sequences. The entire procedure is carried out inside a “black box” until certain aptamers are recognized in the last round [98]. The aptamer selection technology has been transformed by high-throughput sequencing technology, making the selection procedure analyzable in every selection round. Bioinformatics and high-throughput sequencing combined with SELEX have advanced the process of aptamer identification and have given extensive insights into the complete molecular process, thereby providing millions of sequencing data sets from each round of selection. They consist of total reads, primary sequences, nucleotide composition, frequency, and a molecular enrichment rate [99,100]. Because of the evaluation of the library in every round of the selection, high-affinity aptamers can be detected at a much earlier stage, making the procedure inexpensive and eliminating PCR biases related to over selection [98]. 

#### 4.1.4. Techniques to Monitor SELEX Rounds 

Procedures to monitor SELEX rounds can be divided into two main groups. In the first group, affinity estimation of the oligonucleotide is done in every round. Therefore, measurement of binding/dissociation constant of the oligonucleotide library is done by utilizing enzyme-linked oligonucleotide assay (ELONA), surface plasmon resonance (SPR), fluorescent dye-linked aptamer assay (FLAA) that are based on the nature of target molecules and the future use of aptamers [101,102]. The selection process is terminated at the point when the bulk affinity of the library has stopped to further increase or has reached to an expected extent. To measure the pool affinity, to measure the quantity of target-bound RNA/DNA, after every selection cycle is done by using quantitative PCR (qPCR) or fluorescent labeling of the oligonucleotide library. Specific affinity-assessing procedures are also developed for cell-SELEX such as fluorescent imaging, flow cytometry, fluorescent-activated cell sorting (FACS), enzyme-linked modifications oligonucleotide assays, and qPCR adaptations [103,104,105,106,107,108,109]. In the second group, selection progress is assessed by monitoring pool enrichment. In this process, initial highly diverse oligonucleotide pool undergoes evolution under selection pressure and unbound sequences disperse by selection process. In this way, the library’s diversity is decreased by each selection cycle and selection reach to an end when the pool diversity pauses to change. Different methods being used to track the change in diversity include nuclear magnetic resonance (NMR), melting, and reMelting curve analysis of the amplified DNA pool and denaturing HPLC-based techniques [110,111,112,113,114].

Along with this, high-throughput sequencing (HITS) could also be used for evolution tracking [115,116,117,118]. In some studies, a performance comparison was made to monitor the SELEX progress. Five different methods were compared to monitor the SELEX progress by utilizing streptavidin immobilized on magnetic beads as a model target by Mencin et al. [117]. For assessing oligonucleotide pool diversity and for quantification of target bound ssDNA, fluorescent labeling and qPCR used as affinity trackers, fragment length polymorphism analysis and melting curve analysis was done. These analyses were then verified, and comparison was made via the measurement of the K_d_ value of the bulk of DNA pools and for few pools, Sanger sequencing was used. All procedures exhibit subtle correlation with each other and were consistent with the selection outcomes identified by K_d_ measurements and sequencing.

#### 4.1.5. Limitations of SELEX

While establishing a SELEX protocol in a laboratory, various parameters should be kept in consideration like: (i) selection of oligonucleotide type (RNA or DNA), (ii) availability of aptamer preparation equipment, (iii) purification and testing depending upon specific SELEX modification requirement, (iv) nature of target and elution strategy, (v) selection of post-SELEX or in-SELEX modifications, (vi) expected cost and time estimation for the chosen protocol. There is no ideal type of SELEX protocol so far, so researchers should choose different SELEX modifications according to the need and each modification has its limitations. Foremost, the aptamer selection cost should be taken into account. To initiate a selection scheme, massive oligonucleotide library should be constructed by a robotic chemical station in a huge amount [119]. Other option could be purchasing a commercially constructed library, but this will increase the cost of aptamer selection to very high.

Various SELEX modifications have been introduced to reduce the time for aptamer selection, for example, continuous-flow magnetic activated chip-based separation (CMACS) device or micromagnetic separation (MMS) chip [120]. However, devices for automation of each step of SELEX have not been available yet. So, researchers should do all of the steps manually, which is a laborious process. Another major step in the SELEX process is the affinity of aptamers. Various modified SELEX protocols provide different affinity values with K_d_ value range from pM to mM concentrations [121,122,123]. The SELEX strategy that produces the aptamers with K_d_ value at pM and nM concentrations could be a superior choice when time and cost are taken under consideration. In the case of target immobilization on a solid matrix, counter selection helps to remove nonspecific aptamers attached to the target molecules. In addition, when working on structurally alike molecules, counter-selection enables to reduce the cross-reactivity. The counter selection process may also considerably improve the aptamers that are used as therapeutics, consequently minimizing the side effects as a result of nonspecific binding of aptamers to structurally related molecules [124].

## 5. Metabolism and Pharmacokinetics of Therapeutic Aptamers 

### 5.1. Nuclease-Driven Degradation

When unmodified aptamers were studied as therapeutics for systemic administration, three main obstacles related to drug metabolism and pharmacokinetics came up: metabolic instability, fast renal elimination by glomerular filtration, and fast distribution from the blood plasma compartment into tissues [125]. Usually, these characteristics are the same for all aptamer categories, but specific pharmacokinetic hurdles depend upon their category. For a high therapeutic potential, an aptamer should stay for an extended period in blood plasma. To this end, the aptamer needs to be modified to enhance metabolic durability, to slow the exit from plasma, and to retard the removal by renal filtration. Nucleic acid aptamers, including RNA and DNA aptamers quickly degrade outside the cell when nucleases are present, and component nucleotides are metabolized by pyrimidine and purine pathways [126].

### 5.2. Modified Nucleotide Bases

To increase the nuclease resistance and metabolic stability of oligonucleotide aptamers, diverse chemical modifications have been documented and employed. Two techniques, named “in-SELEX” and “post-SELEX,” are utilized to introduce nucleotide modifications into aptamers. 

#### 5.2.1. In-SELEX Technique

In the “in-SELEX technique,” aptamers with appropriately selected modifications are isolated from an RNA or DNA library containing modified nucleotides that are compatible with an RNA or DNA polymerase [127,128]. Nevertheless, this method allows for only a few modifications. The most common modifications of aptamers that are being utilized in clinical studies involve replacing the amino (NH_2_) or *O*-methyl (OCH_3_) group at the 2′ position with a fluorine atom (F) and capping the 3′ end with inverted thymidine to enhance nuclease resistance and binding affinity. To date, 2′-fluoropyrimidines, 2′-*O*-methylnucleotides [129], 2′-aminopyrimidines [130], and locked nucleic acids (LNAs) [131] have been successfully incorporated into SELEX protocols [132]. The first aptamer drug, ‘Macugen’, which is approved to treat age-related macular degeneration is one of the examples of the post-SELEX chemical modification [133].

#### 5.2.2. Post-SELEX Techniques

In post-SELEX, modified nucleotides are inserted at various positions such as the base, sugar ring, phosphate group, or the 2′ position during solid-phase chemical synthesis of aptamers. Sometimes, various modifications are done collectively for maximum efficacy and stability of the aptamer. Because specificity and affinity are related to the structure of aptamers, it is essential to insert the modifications accurately for obtaining desired results, because there is always a risk that affinity and specificity may be reduced [134,135]. There are no universal rules for the modification, and therefore strenuous assessment and optimization are usually required [136]. For instance, if the target molecules have acidic nature, then a positively charged group can be introduced and similarly, for hydrophobic targets, hydrophobic groups are added [135]. Additionally, to generate biostable aptamers, there is one more strategy called “Spiegelmer technology.” Mainly, Spiegelmers are L-RNA (levorotatory RNA) generated from L-ribose units (L-deoxyribose units in the case of DNA) and are non-superimposable mirror images of the natural dextrorotatory forms (D-forms) of the nucleic acid nucleotide monomers. Because of the mirror image property of Spiegelmers, it showed increased resistance to nuclease degradation retaining the affinity for their targets. Moreover, stem-loop modification of aptamer structure has also been used to boost the binding affinity of aptamers for the target molecule [137].

### 5.3. Renal Filtration

The average diameter of 6–30 kDa aptamers falls into the range of size less than 5 nm [138]. When unmodified aptamers enter blood circulation, small aptamers undergo fast excretion through renal filtration. To prevent this fast excretion and to prolong the circulation period in blood plasma, aptamers are usually conjugated with a carrier such as high-molecular-weight polyethylene glycol (PEG) [129], a protein, an organic or inorganic nanomaterial [70,139], or cholesterol [140,141] or are multimerized to obtain a large enough molecule that has a molecular mass higher than the renal glomerulus threshold (30–50 kDa). PEG is a well-known hydrophilic biomolecule that increases the solubility of the conjugated compound and reduces aggregation and enhances in vivo *bioavailability* of therapeutic aptamers after intravenous administration. It is extensively used in Food and Drug Administration (FDA)-approved drugs to increase their circulation half-life. PEGylation of an FDA-approved aptamer, Macugen, expands its half-life from 9.3 to 12 h after subcutaneous or intravenous injection and even to 94 h in the vitreous humor [142]. Monovalent aptamers are the single aptamer molecules, while multivalent aptamers consist of two (dimer) or more (multi) different or identical aptamer motifs that may or may not have some additional structural units [143]. Straightforwardly, joining identical aptamers will enhance the binding affinity because contact points will be increased. While joining the different aptamers will also increase the versatility of aptamers [144]. It is proven that compared to monovalent aptamers, multivalent designed aptamers have shown better efficiency, specificity, binding affinity, circulation time, and biological functions [145]. 

### 5.4. Toxicity 

Toxicity-related data on aptamers in relation to humans are very scarce [146,147]. On the other hand, aptamer-related adverse events have been rare in clinical studies so far. Toxicity-related issues may come up due to unexpected tissue accumulation, high levels of chemical modifications and conjugation, polyanionic effects, or any nonspecific immune activation by repeated doses or continuous dosage of an aptamer therapeutic. Highly harmful charged molecules such as nucleic acids are prone to binding blood proteins nonspecifically, thereby increasing uptake by off-target organs or tissues and resulting in a lower therapeutic potential with specific adverse effects [148,149]. Chemical modifications can have toxic effects or somehow give rise to immunogenicity because the modified nucleotides are unnatural. For instance, hepatotoxicity of LNA-modified nucleic acids has been reported [150]. Therefore, chemical modifications should be selected carefully. Adverse effects can also occur due to specific composition of a therapeutic aptamer. For instance, severe allergic reactions to the PEG group, owing to the presence of preformed antibodies against PEG, have been documented in a phase III trial of Regado Biosciences’ aptamer targeting the anticoagulation system. PEGylated compounds should be used with extra caution in patients with acute diseases [142,151]. 

## 6. Aptamers as Therapeutic Agents

Aptamers acquire almost a similar specificity and affinity as monoclonal antibodies; rather, it has some edge over antibodies. It is hard to produce monoclonal antibodies that are non-immunogenic; however, aptamers are not recognized by the immune system as foreign bodies and do not activate immune response due to the small size of about 30 kDa. The very first aptamer Macugen approved by FDA for the treatment of wet age-related macular degeneration, exhibit no immunogenicity in pre-clinical, animal, or even patient trials [152]. Aptamers with diverse desired properties can be selected, which makes them much suitable as therapeutic agents. RNA and DNA aptamers bind to their target molecules with a dissociation constant (K_d_) within a picomolar or even nanomolar range. Binding of aptamers to the target molecules is the most specific association, and they can distinguish between related proteins that have even the same structural domains. Although binding capacities of antibodies and aptamers are in the same range, aptamers have many extra properties that make them superior to antibodies in various cases. They have high chemical stability for storage and transport, are cleared rapidly, are less immunogenic due to their small molecular size, have much lower dissociation constants ranging from nanomolar to femtomolar values, and are produced without batch-to-batch variation. They can be easily modified to enhance their stability and pharmacokinetic properties. Aptamers are nonimmunogenic at therapeutic doses owing to their similarity to endogenous compounds.

Consequently, aptamers are thought to be an auspicious class of therapeutic and diagnostic candidates and have been widely utilized in imaging, target validation, and therapeutics [153,154]. With improvements of the SELEX technique, various aptamers have been fabricated that specifically target immune-system–related substances, such as chemokines, cytokines, and adhesion and costimulatory molecules [155,156]. In the text below, we overview recently developed aptamers related to the treatment of immune-system disorders.

### 6.1. Aptamers Related to Cell Surface Proteins

TLRs are a group of transmembrane receptors that play a significant part in the defense of the body against invading microorganisms by identifying conserved regions called PAMPs [157]. However, few TLRs, for example, TLR3, 7, 8 and 9 are endosomal and they are displayed on the endosome membrane [158]. To phagocytose these pathogenic microorganisms, various cytokines and chemokines including interleukins and tumor necrosis factor α (TNF-α) are released [159]. TLRs are responsible for triggering various cell signaling pathways to control certain immune responses, for example, MAPK and NF κB pathways [160]. So far, various aptamers have been produced for different TLR-related responses. 

The AP177 aptamer was obtained by the SELEX technology and is a TLR2 antagonist able to inhibit the NF κB activity by 90% induced by TLR1/TLR2 heterodimer, PAM3CSk4 44% NF-kB activity caused by a TLR2/TLR6 heterodimer agonist, FSL-1) [161]. 

Activation of TLR9 initiates a specific immune response in the human body. R10-60 is an immunostimulatory aptamer, and its phosphodiester-modified form is PO R10-60, which has TLR9-agonistic properties. PO R10-60 has a sufficient immunostimulatory effect to start an antigen-specific immune response with fewer adverse effects; this aptamer also has short half-life and controls the accumulation of TNF-α in serum. PO R10-60 is effective in protecting against a deadly infection in the mouse model [162]. Moreover, after prophylactic intranasal administration of PO R10–60 to mice exposed to pulmonary anthrax, the chances of death were dramatically reduced in comparison to PS ISS-ODN [163]. It is well noted that the aptamers modification is being done to modify the phosphodiester form to phosphorothioate form because thioates have affinity to bind the cell surface protein while phosphodiesters are sensitive to nucleases [164].

Aptamer BL-7040 is thought to be effective in the treatment of Sjögren’s syndrome or other autoimmune syndromes because of its acetylcholinesterase-inhibitory activity after binding to TLR9 [121]. Additionally, two RNA aptamers have been discovered that have high affinity for the TLR3 ectodomain [165]. 

### 6.2. Costimulatory Receptors

OX40 is one of the costimulatory receptors present on CD4^+^ T cells in their activated state and binds to its ligand, OX40-L, which is expressed on antigen-presenting cells. Moreover, 9C7 is an aptamer with high affinity for human OX40 and competes with human OX40-L for the natural binding site on receptor OX40. This aptamer can specifically bind to the receptor and increase interferon γ production and T lymphocyte expansion, reflecting its high potential as a therapeutic agent for cellular therapy [166]. 

An RNA aptamer with noticeable affinity for receptor OX40 activates cytokine production and T-cell proliferation, whereas a DNA aptamer against receptor OX40 functions as an agonist driving the release of cytokines along with the proliferation of leukocytes [167]. 

B lymphocytes express B-cell receptors (BCRs) on their plasma membrane, which activate B cells after binding to certain antigens. A DNA aptamer, TD05, has been selected by the Cell-SELEX technology and has specificity and affinity for the heavy chain of a BCR. After introduction of several modifications such as LNA for increased nuclease resistance and conformational stability, truncation for improved binding, and PEGylation at the aptamer ends for improved stability and pharmacological performance, the TD05 aptamer has high stability at physiological temperature both in vitro and in vivo. Given that a BCR is the primary marker of Burkitt’s lymphomas, it is possible to use TD05 in the early diagnosis and cell-targeted therapy for Burkitt’s lymphomas [168].

### 6.3. Costimulatory Molecules

Costimulatory molecules of T lymphocytes include cytotoxic T-lymphocyte protein 4 (CTLA-4), CD28, programmed death-ligand 1 (PDL1), and inducible costimulator (ICOS). CTLA-4 and CD28 associate with CD86 and CD80 to modulate the properties of T cells bound to the membrane of antigen-presenting cells [169]. Minimizing the binding of CD28 to B7 may be a promising approach to reducing allograft rejection [170,171].

STAT3 is the main factor promoting tumor growth by activating CD4^+^ regulatory T cells, which ultimately suppress CD8^+^ cytotoxic T cells and enhance tumor growth. The affinity of CTLA4apt (aptamer to CTLA4) to STAT3 small interfering RNA (CTLA4apt-STAT3 siRNA) may sharply decrease the number of tumor-associated CD4^+^ regulatory Tregs. The CTLA4apt-STAT3 siRNA effectively boosts antitumor immune responses and slows tumor growth by lowering STAT3 activation in mouse models [172]. CD28-agonistic aptamers block the B7–CD28 interaction, thereby reducing costimulatory signals and lymphocyte proliferation and ultimately inactivating lymphocytes. Two 2-fluoropyrimidine-modified RNA aptamers against CD28 (CD28Apt7 and CD28Apt2) have been chosen in a murine model. The CD28Apt2 monomer efficiently blocks the B7.2 interaction, which leads to a reduction in the proliferation of purified CD4^+^ lymphocytes. Dimeric CD28Apt7 has manifested a more substantial proliferative effect on murine CD4^+^ lymphocytes as compared to CD28Apt2-dimer. Nevertheless, CD28Apt2-dimer causes the same outcome as do anti-CD28 antibodies. In the dimer form, the aptamer has a more substantial costimulatory effect, shorter linker size, and lower therapeutic doses and toxicity. The CD40 aptamer and CD28 aptamer form bispecific aptamers and thus show enhanced efficacy in the treatment of allogeneic-transplant rejection [173]. 

### 6.4. Aptamers for the Complement System

The complement system contains a highly organized network of ~30 proteins that perform crucial functions defense system of host against inflammation. These proteins are usually present in the blood in a soluble, inactive precursor state and are activated by different triggers that initiate a tightly regulated cascade of enzymatic reactions [174]. All of these enzymatic reactions result in the production of a membrane attack complex on the cell membrane, creating intramembrane holes leading to cell death [175]. An active form of complement component C5 is C5a, which plays an essential part in neutrophil stimulation and ends up in the release of proinflammatory cell mediators that cause organ damage. The ARC1905 aptamer inhibits the activation of C5 to the functional form, C5a. The NOX-D20 aptamer effectively blocks C5a-induced cellular responses by competing with the C5a receptor for binding to C5a, thus improving the symptoms in experimental sepsis model [176]. Additionally, the MUC1-5TR-1 aptamer is linked to a complement component C fragment, C1q, and triggers the classical complement system pathway, which raises cell mortality [177]. 

### 6.5. Aptamers for Cytokines 

Cytokines modulate host responses against infection, trauma, and inflammation [178]. Different classes of cytokines such as chemokines, interleukins, interferons, transforming growth factors (TGFs), and TNFs engage the corresponding receptors and trigger downstream cell signaling pathways to regulate various immune responses [179,180]. An excess release of cytokines produces exaggerated systemic inflammatory reactions that cause multiorgan failure, autoimmune diseases, and graft-versus-host disease. Until now, various cytokines have been utilized as therapeutic agents. For example, erythropoietin (EPO) is used to treat anemia, granulocyte colony-stimulating factor (G-CSF) against neutropenia, and granulocyte-macrophage colony-stimulating factor (GM-CSF) to treat neutropenia and fungal infections. Moreover, bone morphogenetic protein (BMP) is employed to treat bone diseases, IL-11 to treat thrombocytopenia, and interferon to treat sclerosis [181,182]. Hence, various aptamers have been produced to enhance or decrease cytokine action. 

Interleukins are a class of cytokines that promote the activation and differentiation of hematopoietic cells and B and T lymphocytes [183]. IL-8 initiates phagocytosis and chemotaxis of neutrophils to an infection site and it is also involved in pathogenesis of cancers. Aptamer 8A-35 has an agonistic potential for neutralizing the IL-8 activity and has therapeutic effects against various inflammatory diseases [184]. 

RNA aptamers assist the IL-10 receptor (IL-10R) to suppress tumor growth and viral infection [185]. IL-6R plays an essential role in numerous inflammatory diseases, including pristane-induced lupus, plasmacytomas, cancer metastases, myeloma, and arthritis. AIR-3A is an RNA aptamer that has therapeutic potential against IL-6R–related disorders [186,187]. Another aptamer, SL1026, is an antagonist of IL-6 and inhibits IL-6 signaling in vitro. SL1026 postpones the outset and lessens the severity of rheumatoid arthritis symptoms in a cynomolgus monkey model of collagen-induced arthritis [188].

Interferon γ is a glycoprotein secreted by lymphocytes and has antiviral, antitumor, and immunomodulatory functions. A novel modified DNA aptamer, 57 mhGC, targeting interferon γ, holds promise for therapeutics and diagnostics [189].

TNFα is a vital component of the cytokine network and plays an essential part in inflammatory diseases. VR11 is a short single-stranded DNA aptamer with high affinity for human TNFα and inhibits TNFα signaling significantly with a dissociation constant of 7.0 ± 2.1 nM [190]. Innate immune system related aptamers are enlisted in Table 1.

## 7. Concluding Remarks

Aptamers are single-stranded oligonucleotides that have specificity and affinity for their target molecules. We explained the production and pharmacokinetics of aptamers and reviewed the aptamers that have been discovered for innate-immunity–related targets. This new class of biotherapeutics gained much attention in recent years. Many therapeutic aptamers have made headway to the mid or late stages of clinical trials and have manifested remarkable characteristics for local and systemic applications. The main obstacle to the development and use of aptamers in diagnostics and biotherapeutics is the heavy investment of pharmaceutical and biotech industries in the well-established antibody and humanized monoclonal antibody research, development, and manufacturing. This state of affairs makes these industries hesitant to finance new therapeutics such as aptamers. Regardless of these challenges, the superiority of aptamers to antibodies has led to some market penetration. Thus, the aptamer technology has a bright future in therapeutics and diagnostics. In the next 5 to 10 years, we forecast many aptamer-based therapeutic and diagnostic products carving out their niches in the vast worlds of biopharmaceutics and diagnostics.

## Figures and Tables

**Table 1:** Innate-immune-system–related aptamers.

Name of Aptamer	Target	Nature of Aptamer	Clinical Phase	Chemical Modification	References
PO R10-60	TLR9	RNA		phosphodiester	[163,191]
MP7	(PD1)	DNA	Preclinical	unmodified	[192]
SDA	E-selectin and P-selectin	DNA	Preclinical	unmodified	[193]
ESTA-1	E-selectin	DNA	Preclinical	only one thio-modified nucleotide	[193]
9C7	OX40	RNA	Preclinical	Modified initial 2-fluoro (2-F)	[166,167]
MUC1-5TR-1	C1q	DNA	Preclinical	5- Alexa Fluor 546 (AF546) or 5′-biotin modifications	[177]
8A-35	IL-8	RNA	Preclinical	2′-fluoro-pyrimidine modified	[184]
AIR-3	Interleukin 6 receptor	RNA	Preclinical	Shortening of FAIR-6 or 2′-F-Py modified AIR-3	[194,195,196]
SL1026	Interleukin 6	RNA	Preclinical	a hexylamine modification	[155]
ARC1950	Human complement C5	RNA	Phase 2	2’-O-methyl purine and 2’-fluoropyrimidine substitutions	[197]
AS1411	Nucleolin	DNA	Phase 2	unmodified (phosphodiester) DNA backbone	[198]
NOX A12	CXCL12	RNA	Phase 2	L-configuration containing branched 40 kDa PEG	[199]
NOX-H94	Hepcidin	RNA	Phase 2	5’ end with 40 kDa PEG tail	[200]
ARC1779	A1 domain of vWF	DNA/RNA	Phase 2	phosphorothioate	[201,202]
NU172	Thrombin	DNA	Phase 2	unmodified 26-nucleotide DNA aptamer	[203]

Abbreviations: C1q, complement component 1q; CXCL12, C-X-C motif chemokine 12; IL-6, interleukin-6; IL-8, interleukin-8; PD1, programmed cell death protein 1; TLR9, Toll-like receptor 9; vWF, von Willebrand factor.

## Abbreviations

AP-1	Activated Protein 1
BCR	B-Cell Receptor
BMP	Bone Morphogenetic Protein
C5	Compliment component 5
CD	Cluster of Differentiation
CLR	C-type Lectin Receptor
CTLA-4	Cytotoxic T Lymphocyte Associated Antigen 4
DAMP	Damage-associated Molecular Pattern
C5	Compliment component 5
CD	Cluster of Differentiation
CLR	C-type Lectin Receptor
CTLA-4	Cytotoxic T Lymphocyte Associated Antigen 4
DAMP	Damage-associated Molecular Pattern
DNA	Deoxy Ribonucleic Acid
ELONA	Enzyme Linked Oligonucleotide Assay
EPO	Erythropoietin
FACS	Fluorescent Activated Cell Sorting
GM-CSF	Granulocyte Macrophage Colony Stimulating Factor
HPLC	High Performance Liquid Chromatography
IFN-γ	Interferon γ
Ig	Immunoglobulin
IKK	Inhibitor of κB kinase
IL	Interleukin
IRAK	Interleukin Receptor Associated Kinase
IRF	Interferon Response Factor
IκB	Inhibitor of κB
K_D_	Dissociation Constant
LNA	Locked Nucleic Acid
LRR	Leucine-Rich Repeat
MAC	Membrane Attack Complex
MAL	MyD88 Adaptor Like
MAP	Mitogen-Activated Protein
MAPK	Mitogen-Activated Protein Kinase
MASP	MBL-Associated Serine Protease
MBL	Mannose-Binding Lectin
MHC	Major Histocompatibility Complex
MyD88	Myeloid Differentiation primary response 88
NEMO	NF-κB Essential Modulator
NF-κB	Nuclear Factor-κB
NLR:	NOD-Like Receptor
NMR	Nuclear Magnetic Resonance
NOD	Nucleotide Oligomerization Domain
PAMP	Pathogen Associated Molecular Pattern
PCR	Polymerase Chain Reaction
PDL1	Programmed Death-Ligand 1
**PEG**	Polyethylene Glycol
PRR	Pattern Recognition Receptor
qPCR	Quantitative PCR
RIP	Receptor Interacting Protein
RNA	Ribonucleic Acid
RT	Reverse Transcription
SELEX	Systematic Evolution of Ligands by Exponential Enrichment
siRNA	Small Interfering RNA
STAT3:	Signal Transducer and Activator of Transcription 3
TAB	TAK1-Binding Protein
TAK1	Transforming Growth Factor β–Activated Kinase 1
TBK1	TANK-Binding Kinase 1
TIR	Toll/Interleukin-1 Receptor
TIRAP	TIR domain-containing Adaptor Protein
TLR	Toll-Like Receptor
TNFR	Tumor Necrosis Factor Receptor
TNFα	Tumor Necrosis Factor α
TRAF	Tumor Necrosis Factor Receptor Associated Factor
TRAM	TRIF-Related Adaptor Molecule
TRIF	TIR-domain-containing Adapter-inducing Interferon-β
C1q	Complement Component 1
CXCL12	C-X-C Motif Chemokine Ligand 12
IL-8	Interleukin-8
vWF	von Willebrand Factor
